# In-plane Isotropic Microwave Performance of CoZr Trilayer in GHz Range

**DOI:** 10.1038/srep21327

**Published:** 2016-02-17

**Authors:** Lulu Pan, Fenglong Wang, Wenfeng Wang, Guozhi Chai, Desheng Xue

**Affiliations:** 1 Key Laboratory for Magnetism and Magnetic Materials of the Ministry of Education Lanzhou University, Lanzhou, 730000, People’s Republic of China

## Abstract

In this paper, we investigate the high frequency performance of Co_90_Zr_10_/SiO_2_/Co_90_Zr_10_ trilayers. It is demonstrated that the in-plane isotropic microwave performance is theoretically derived from the solution of the Landau-Lifshitz-Gilbert equation and experimentally achieved in that sandwich structured film. The valuable isotropic behavior comes from the superposition of two uncouple ferromagnetic layers in which the uniaxial magnetic anisotropic fields are equivalent but mutually orthogonal. Moreover, the isotropic microwave performance can be tuned to higher resonance frequency up to 5.3 GHz by employing the oblique deposition technique. It offers a convenient and effective way to achieve an unusual in-plane isotropic microwave performance with high permeability in GHz, holding promising applications for the magnetic devices in the high frequency information technology.

Soft magnetic materials with isotropic high permeability (IHP) at working frequency are crucial components in modern information technology because of their extensive applications to improve the performance of magnetic devices such as micro-transformers, planar inductors and core materials of writing head^1^,^2^,^3^. Nowadays, data transfer rate is getting to GHz, but the classical Snoek’s law^4^ indicates that the IHP based on crystalline anisotropy in the traditional microwave soft magnetic materials can only be obtained in the MHz range. To find the desired materials with IHP in the GHz range is still a challenge. For instance, a variety of theoretical^5^,^6^,^7^ and experimental works focus on both high^8^,^9^,^10^ and isotropic^11^,^12^,^13^,^14^,^15^,^16^,^17^,^18^ permeability in the GHz range of granular films^9^ and multilayers^10^ of magnetic alloys, ferrites and their composites.

As Kittel predicted in 1947^19^, magnetic thin film is a good candidate to achieve high permeability in the GHz range. The high permeability in the GHz range was measured experimentally in the CoZrNb magnetic thin films with an in-plane uniaxial magnetic anisotropy (IPUMA) in 1996^20^. Subsequently, many film systems of alloys, ferrites and their composites have been investigated^8^,^9^,^21^,^22^,^23^. Therein, metallic magnetic thin films, which have higher saturation magnetization than ferrite films, are better to achieve higher permeability and resonance frequency. Besides the large saturation magnetization *M*_*s*_, the large IPUMA is essential for high permeability in the GHz range^24^. As the frequency response of the permeability is almost flat up to a rolloff frequency, associated with ferromagnetic resonance, when a microwave magnetic field **h** is applied perpendicular to the IPUMA field **H**_*K*_, the resonance frequency *f*_*r*_ and initial permeability *μ*_*in*_ of the magnetic thin films can be adjusted by *H*_*K*_ as 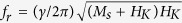
19 with *γ* as the gyromagnetic ratio and 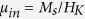
22, respectively. In fact, the IPUMA field **H**_*K*_ has been modulated by many effective methods, such as composition gradient sputtering^25^,^26^, micro-stripe patterning^27^,^28^,^29^,^30^, annealing under magnetic fields^31^,^32^,^33^, inducing stress on substrates^34^,^35^,^36^, oblique deposition^37^,^38^, temperature^39^ and electric field control^40^,^41^, etc. The permeability higher than 100 was achieved in the 1–5 GHz range in (Fe, Co)-based magnetic thin films with the IPUMA^24^. However, the high permeability of magnetic thin films with the IPUMA is anisotropic, which depends on the relative direction of **h** with respect to **H**_*K*_.

Searching for the isotropic permeability in the GHz range is another important topic in magnetic thin films. For instance, magnetic thin films with rotatable stripe domain^42^,^43^,^44^,^45^,^46^, composite-anisotropy multilayer as well as crossed anisotropies multilayer^11^,^12^,^13^,^14^,^15^,^16^,^17^,^18^ are intensively investigated. The rotatable stripe domain was discovered firstly in Permalloy films in 1961^42^, and then in many other magnetic thin films^43^,^44^,^45^,^46^. As the effective IPUMA is along the direction of the stripe domain, the in-plane omnidirectional equivalent permeability can be achieved^46^ by rotating the stripe domain. It is worth noting that this is a spurious isotropic performance, because an external saturated magnetic field needs to be applied to rotate the orientation of the stripe domains which may limit the application of the magnetic thin films in magnetic devices. Similar results are found in other magnetic thin films with rotatable anisotropy^46^. The composite-anisotropy multilayer^11^,^12^,^13^,^14^,^15^ is another idea to search for the isotropic permeability, in which many equivalent magnetic layers having the same IPUMA are piled up. Similar to a random particle composite, the overall anisotropy of the multilayer can be cancelled out by shifting sequentially the anisotropic axis of every layer from bottom to surface^11^. The isotropic permeability is then achieved. Furthermore, the crossed anisotropies multilayers^16^,^17^,^18^ were also investigated for the purpose of isotropic permeability. However, the resonance frequencies of those multilayers are normally lower than 2 GHz. Therefore, seeking for magnetic thin films with the IHP in higher frequency is still expected not only for fundamental research but also for the technological applications.

In this paper, we report the achievement of an in-plane IHP with resonance frequency higher than 5 GHz in a convenient FM1/NM/FM2 (FNF) film, in which two ferromagnetic layers (FM*i*, *i* = 1, 2) are decoupled by a non-ferromagnetic interlayer (NM). By theoretical analysis, FM1 and FM2 with the same magnetic moment and equivalent but mutually orthogonal IPUMA fields *H*_*K*_ are proposed. The isotropic microwave performance with an in-plane IHP is found experimentally in a Co_90_Zr_10_/SiO_2_/Co_90_Zr_10_ FNF film. Moreover, it is indicated that the in-plane IHP characteristics of these FNF films can even be extended to higher working frequency by employing the oblique deposition technique.

## Theoretical analysis

**Figure 1:**
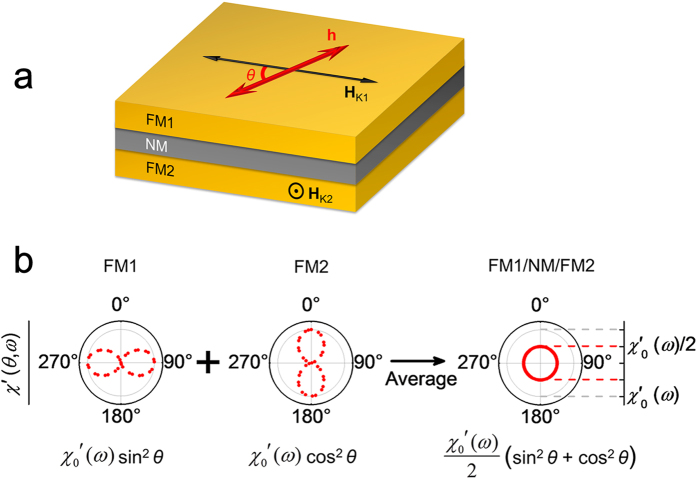
Schematic diagram and the angular dependences of the susceptibility of FM1/NM/FM2 film. (**a**) **H**_*K*1_ and **H**_*K*2_ (**H**_*K*1_ ⊥ **H**_*K*2_, and *H*_*K*1_ = *H*_*K*2_) are the in-plane uniaxial magnetic anisotropy fields of the FM1 and FM2 ferromagnetic layers, NM is the non-ferromagnetic interlayer, and *θ* is the angle between **H**_*K*1_ and the microwave magnetic field **h**. (**b**) *θ* dependences of the susceptibility under **h**, where the left, middle and right polar diagrams are theoretical simulation results of FM1, FM2 and FM1/NM/FM2, respectively.

Figure 1 illustrates the FNF film designed to realize the in-plane IHP in the GHz range, in which the IPUMA fields are perpendicular to each other. Considering a microwave magnetic field **h** applied in the plane of the FNF film as shown in Fig. 1a, the complex susceptibility of the film comes from weighted average of complex susceptibility of FM1 and FM2, written as


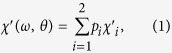



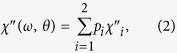


where 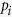 (*i* = 1, 2) is the volume ratio of the FM*i* layer to the two magnetic layers. The microwave susceptibility of each magnetic layer results from uniform precession of magnetization, which can be described by LLG equation^47^, so the susceptibility of FM*i* layer 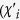 and 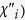 can be derived from LLG equation, and given by


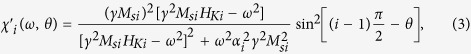



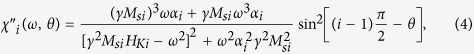


where *γ* is the gyromagnetic ratio, *α*_*i*_ is damping parameter, *ω* is the angular frequency of **h** and *θ* is the angle between **h** and **H**_*K*1_, respectively. As 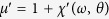 and 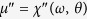, the complex permeability is generally angular dependent. Combining Equation (1), (2), and Equation (3), (4) with the conditions of


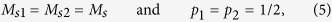



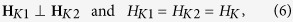



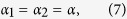


an angular independent complex permeability of the FNF film can be derived as


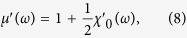



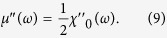


Herein, 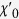 and 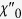 are the real and imaginary parts of the complex susceptibility of a single FM layer under the condition 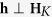, as^48^


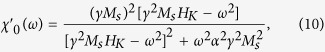



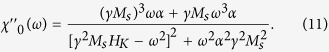


Consequently, the in-plane IHP can be realized in the FNF film as shown in Fig. 1(b).

## Results and Discussion

In experiments, Co_90_Zr_10_(100 nm)/SiO_2_(10 nm)/Co_90_Zr_10_(100 nm) FNF films with in-plane IHP at GHz were fabricated by radio-frequency magnetron oblique sputtering (see Supplementary Fig. S1 online). Here Co_90_Zr_10_ (EDS and XRD data are shown in Supplementary Fig. S2 online) used as FM layer, which possesses a relative high saturation magnetization and a well-established IPUMA^49^,^50^, can result in the high permeability at GHz. Each Co_90_Zr_10_ layer was about 100 nm to ensure that *M*_*s*1_ = *M*_*s*2_ = *M*_*s*_ and *p*_1_ = *p*_2_ = 1/2. A 10 nm SiO_2_ interlayer was chosen to eliminate the exchange coupling between two FMs, and thus the IPUMA of each Co_90_Zr_10_ layer can be adjusted separately. The direction and strength of the IPUMA were induced by controlling the oblique deposition conditions to satisfy **H**_*K*1_ ⊥ **H**_*K*2_ and *H*_*K*1_ = *H*_*K*2_, *α*_1_ = *α*_2_ = *α* as the two Co_90_Zr_10_ layers are equivalent. The details are discussed in the supplementary information.

### Static magnetic performance

**Figure 2:**
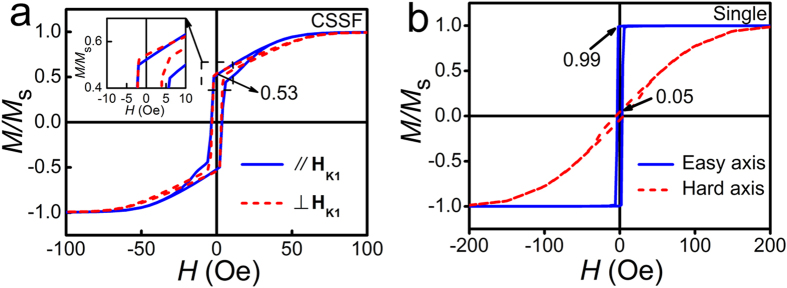
In-plane magnetic hysteresis loops of the films fabricated by 30° oblique deposition. (**a**) Loops of the Co_90_Zr_10_/SiO_2_/Co_90_Zr_10_ FNF film. The blue solid line and red dashed line represent the curves measured along and perpendicular to the anisotropy field **H**_*K*1_. The insert is an enlarged view of the loops near the remnant ratio 0.53. (**b**) Loops of the Co_90_Zr_10_ single layer. The blue solid line and red dashed line represent the curves measured along easy and hard axis, in which the remnant ratios are 0.99 and 0.05, respectively.

Figure 2 depicts the in-plane magnetic hysteresis loops of the Co_90_Zr_10_ FNF film fabricated by 30° oblique deposition, as well as that of a Co_90_Zr_10_(100 nm) single layer as a comparison. Two significant characteristics of the loops shown in Fig. 2a indicate that the conditions for achieving IHP are met in the Co_90_Zr_10_ FNF film. Shape of the loops in Fig. 2a, which is different from that in Fig. 2b, can be explained as the superposition of loops along the easy and hard axis of FM, respectively. The loops parallel and perpendicular to the direction of **H**_*K*1_ are almost identical, which implies the saturation magnetization, as well as the anisotropies, of FM1 and FM2 are equivalent, i.e., *M*_*s*1_ = *M*_*s*2_ and *H*_*K*1_ = *H*_*K*2_. To clarify the results above, the remanence and the slope near *H* = 0 Oe are discussed further in the following. The remanence ratio of 0.53 shown in Fig. 2a is the weighted average of the remanence ratios 0.99 and 0.05 obtained from the easy and hard axis loops in Fig. 2b, that means **H**_*K*1_ ⊥ **H**_*K*2_. Moreover, the equal remanence *M*_*ri*_ (*i* = 1, 2) ≈ *M*_*si*_/2 along **H**_*Ki*_, indicates *M*_*s*1_ = *M*_*s*2_. Meanwhile, the same slope at *H* = 0 Oe for both loops, which can be expressed as *M*_*s*_/*H*_*K*_, reveals *H*_*K*1_ = *H*_*K*2_ as well. As the Co_90_Zr_10_ FNF film satisfies the conditions for achieving IHP very well, it is expected to have in-plane IHP as predicted by Equation (8)–(11), 16, 20, 21. It should be noted that the hysteresis loops of FNF trilayer are not fully isotropic even the permeability of the FNF is angular independent.

### Dynamic magnetic performance

**Figure 3:**
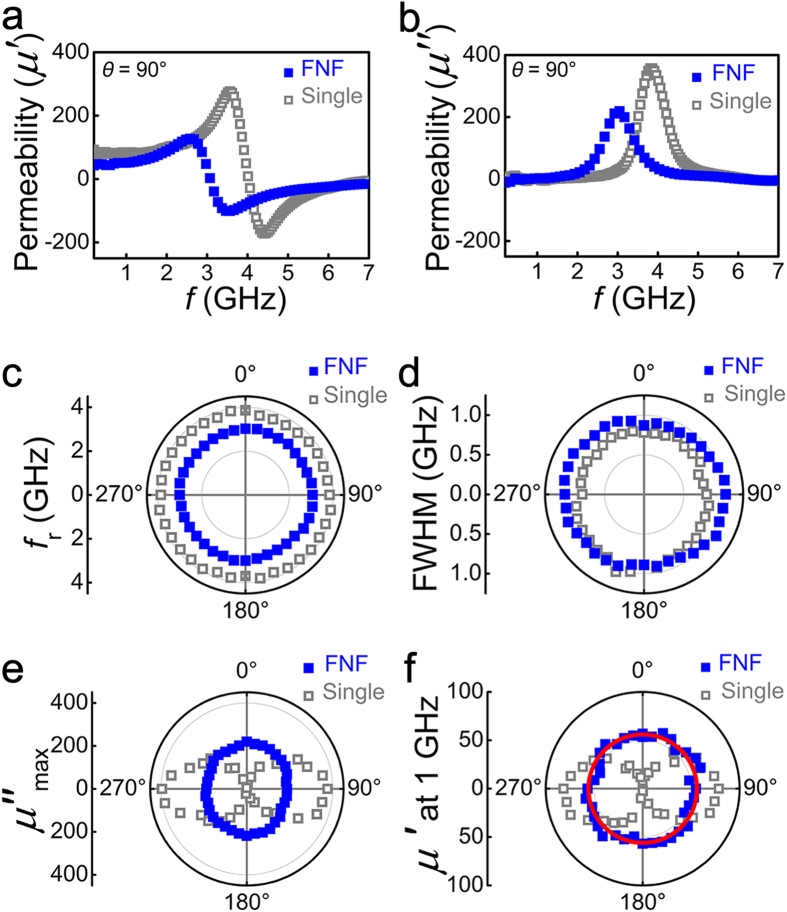
GHz frequency characteristics of the Co_90_Zr_10_ FNF film (blue solid squares) and the Co_90_Zr_10_ single layer (grey open squares) fabricated by 30° oblique deposition. (**a**) Real and (**b**) imaginary parts of permeability spectra measured at *θ* = 90°. (**c**) Resonance frequency, (**d**) the FWHM, and (**e**) *μ*″ maximum as a function of *θ*. (**f**) Real part of permeability at 1 GHz as a function of *θ*. The red solid line is theoretical simulation result of Co_90_Zr_10_ FNF film.

The microwave performances of the Co_90_Zr_10_ FNF film and a Co_90_Zr_10_ single layer are shown in Fig. 3. Figure 3(a,b) displays a typical frequency dependence of the real (imaginary) part of the sample permeability *μ*′ (*μ*″) measured at *θ* = 90°. The real part *μ*′ higher than 50 is obtained from 1.0 to 2.5 GHz. The fitting results indicate both the Co_90_Zr_10_ FNF film and the single layer exhibit a resonance-type permeability spectra, i.e. the spectrum of the real (imaginary) part is a dispersive (Lorentzian) curve. In order to demonstrate the isotropic microwave properties, the angular dependence of the resonance frequency, the maximum value and the full width at half maximum (FWHM) of the imaginary parts are plotted in Fig. 3c–e, respectively. The angular independence of the three characteristic quantities of the Lorentzian curves indicates that the imaginary part of the Co_90_Zr_10_ FNF film is isotropic. Based on the Kramers-Kronig relation, the real part of the Co_90_Zr_10_ FNF film must be also isotropic, which is confirmed by the experimental data shown in Fig. 3f, where the angular dependence of the permeability *μ*′ at 1 GHz of the Co_90_Zr_10_ FNF film shows clearly a circular characteristic of the IHP instead of a spindle-like distribution for the Co_90_Zr_10_ single layer. By fitting the data in Fig. 3f, the IHP at 1 GHz is around 55 for the Co_90_Zr_10_ FNF film. Those results reveal that the isotropic microwave performance with the IHP is achieved in the Co_90_Zr_10_ FNF film rather than in the Co_90_Zr_10_ single layer.

**Figure 4:**
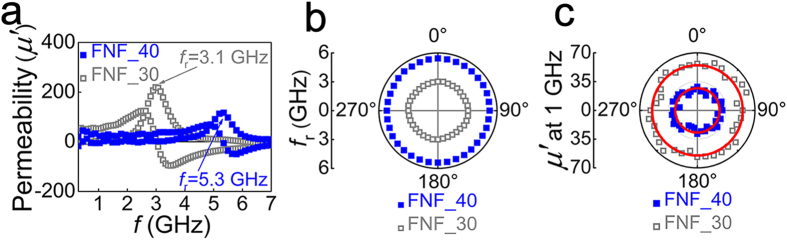
GHz frequency characteristics of the Co_90_Zr_10_ FNF films fabricated by 40° (blue solid squares) and 30° (grey open squares) oblique deposition. (**a**) permeability spectra. (**b**) *θ* dependence of resonance frequency *f*_*r*_. (**c**) *θ* dependence of permeability (*μ*′) at 1 GHz. The red solid lines are theoretical simulation results.

In order to extend experimentally the working frequency range of the Co_90_Zr_10_ FNF film with IHP and exploit the universality of the IHP of the FNF film, the Co_90_Zr_10_ FNF film with the same structure as previous but higher resonance frequency was fabricated by 40° oblique deposition. According to the self-shadow effect^51^,^52^,^53^, the larger angle of the oblique deposition leads to the larger IPUMA field *H*_*K*_ of Co_90_Zr_10_ single layer and consequently the higher resonance frequency. The tunable high frequency properties of oblique deposited Co_90_Zr_10_ single layer can be found in Fig. S3 or in our previous works^49^,^50^. The isotropic microwave performances of this Co_90_Zr_10_ FNF film are displayed in Fig. 4, together with the corresponding data of 30° oblique deposition Co_90_Zr_10_ FNF film. Figure 4a shows typical frequency dependence of the real part of permeability for both Co_90_Zr_10_ FNF films. With the oblique deposition angle increasing from 30° to 40°, the *f*_*r*_ of the Co_90_Zr_10_ FNF film increases from 3.1 to 5.3 GHz. Figure 4b shows *f*_*r*_ vs *θ* plots, where the circular distribution implies the isotropic resonance frequency. The isotropic effective fields are calculated from the resonance frequency as about 299 Oe for Co_90_Zr_10_ FNF film fabricated by 40° oblique deposition and 102 Oe for the 30° oblique deposition sample. Figure 4c shows *μ*′ at 1 GHz vs *θ* plots of the two Co_90_Zr_10_ FNF films as well as the fitted curves with Equation (8). The high frequency permeability always keep isotropic even the resonance frequency has been pushed from 3.1 to 5.3 GHz. These results reveal that the working frequency range of the Co_90_Zr_10_ FNF film with in-plane IHP can be adjusted by tuning the oblique deposition angle.

In conclusion, we have demonstrated that the Co_90_Zr_10_(100 nm)/SiO_2_(10 nm)/Co_90_Zr_10_(100 nm) sandwich-structured films fabricated by the oblique deposition exhibit an isotropic microwave performance, and especially an isotropic high permeability larger than 20 with resonance frequency up to 5.3 GHz. The valuable isotropic behavior comes from the superposition of two uncouple equivalent ferromagnetic layers in which the uniaxial anisotropic fields are mutually orthogonal. The finding of the in-plane isotropic high permeability with high working frequency of the universal FM/NM/FM sandwich-structured film may benefit in searching for new microwave materials and have important applications in magnetic devices desired in the information technology.

## Methods

### Sample fabrication

The Co_90_Zr_10_(100 nm)/SiO_2_(10 nm)/Co_90_Zr_10_(100 nm) sandwich-structured films and Co_90_Zr_10_(100 nm) single layer were deposited at room temperature onto Si (111) substrates by radio frequency magnetron sputtering, and the in-plane uniaxial magnetic anisotropy of each Co_90_Zr_10_ layer was induced by oblique deposition. The base vacuum is 8.5 × 10^−5^ Pa, the sputtering power is 50 W, the flow of Ar is 10 SCCM, and the sputtering pressure is 0.25 Pa. The component of Co_90_Zr_10_ layer is tuned by putting a few Zr chips on the Co target, and the thickness of each layer was controlled by deposition time and rate. The Co_90_Zr_10_ FNF film is deposited as follows: the Co_90_Zr_10_(100 nm) bottom layer on Si substrate is deposited firstly, after turning the sample by 90 degrees on the sample holder, SiO_2_(10 nm) interlayer and the Co_90_Zr_10_(100 nm) top layer were deposited, successively.

### Measurement

The composition of the films was determined by energy dispersive X-ray spectroscope (EDS) and the structure of the films was characterized by an X-ray diffractometer. A vibrating sample magnetometer (VSM) was employed to measure the hysteresis loops of the samples at room temperature. The remanence depending on angle of the Co_90_Zr_10_ FNF film was used to determine firstly the directions of **H**_*K*1_ and **H**_*K*2_, and then in-plane magnetic hysteresis loops were measured along **H**_*K*1_ and **H**_*K*2_. The similar measurement procedure was used for the Co_90_Zr_10_ single layer film as well. In order to get permeability spectra at different *θ*, we used vector network analyzer (VNA) with shorted micro-strip method^54^. The isotropic effective field can be calculated from the resonance frequency with equation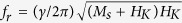, but considering *H*_*K*_≪ *M*_*s*_ and for simplicity, 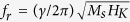 was used to calculate the effective field in this paper.

## Additional Information

**How to cite this article**: Pan, L. *et al*. In-plane Isotropic Microwave Performance of CoZr Trilayer in GHz Range. *Sci. Rep*. **6**, 21327; doi: 10.1038/srep21327 (2016).

## Supplementary Material

Supplementary Information
