# Supplementary material for: In-plane Isotropic Microwave Performance of CoZr Trilayer in GHz Range
Source: Sci Rep. 2016 Feb 17;6:21327. doi: 10.1038/srep21327 (PMC4756334; doi:10.1038/srep21327)
Supplement: Supplementary Information [file srep21327-s1.doc]

Supplementary Information:

**In-plane Isotropic Microwave Performance of CoZr Trilayer in GHz Range**

**Lulu Pan**^1^**, Fenglong Wang**^1^**, Wenfeng Wang**^1^**, Guozhi Chai**^1^* **and Desheng Xue**^1^*

1 Key Laboratory for Magnetism and Magnetic Materials of the Ministry of Education Lanzhou University, Lanzhou, 730000, People’s Republic of China

* E-mail: [chaigzh@lzu.edu.cn](mailto:chaigzh@lzu.edu.cn), [xueds@lzu.edu.cn](mailto:xueds@lzu.edu.cn)

S1. Fabrication of the Co_90_Zr_10_ layer and controlling the direction of anisotropy


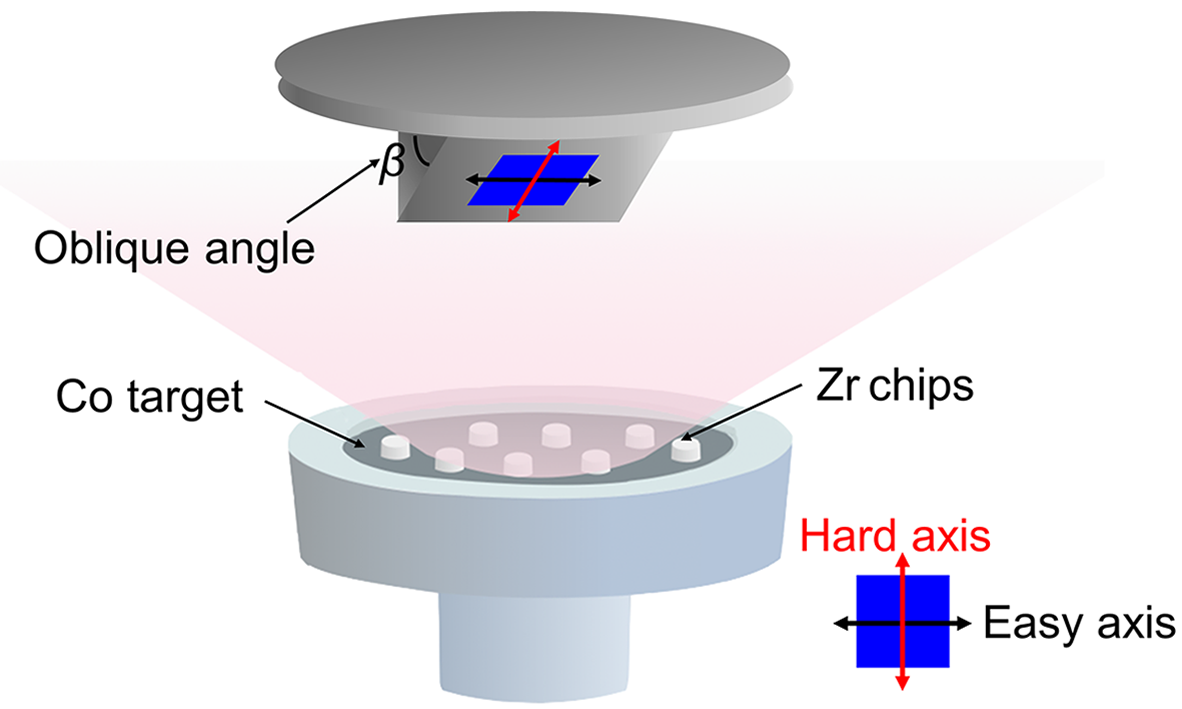


**Supplementary Figure S1.** Sketch of oblique deposition of the Co_90_Zr_10_ layer. *β* is the oblique angle, the blue square on the sample holder is the Co_90_Zr_10_ layer on Si(111) substrate. The insert at bottom right corner shows the direction of easy axis (black line) and hard axis (red line) of the Co_90_Zr_10_ layer.

As discussed in the main text, the anisotropy of each Co_90_Zr_10_ layer is induced by oblique deposition and satisfy **H***_K_*_1_ ⊥ **H***_K_*_2_. Supplementary Fig. S1 shows the sketch of oblique deposition of the Co_90_Zr_10_ layer that was employed in this work. By this method, the direction of anisotropy of each Co_90_Zr_10_ layer is tuned, the insert at bottom right corner shows the direction of easy axis (black line) and hard axis (red line) of the Co_90_Zr_10_ layer. After the Co_90_Zr_10_ (100 nm) bottom layer was deposited on Si(111) substrate firstly, then turned the sample 90 degrees on the sample holder, SiO_2_(10 nm) interlayer and the Co_90_Zr_10_(100 nm) top layer were deposited, successively. By that procedure, the anisotropies of the two Co_90_Zr_10_ layers are perpendicular to each other, i.e. **H***_K_*_1_ ⊥ **H***_K_*_2_.

S2. Determination of the structure and the composition of the Co_90_Zr_10_ layer


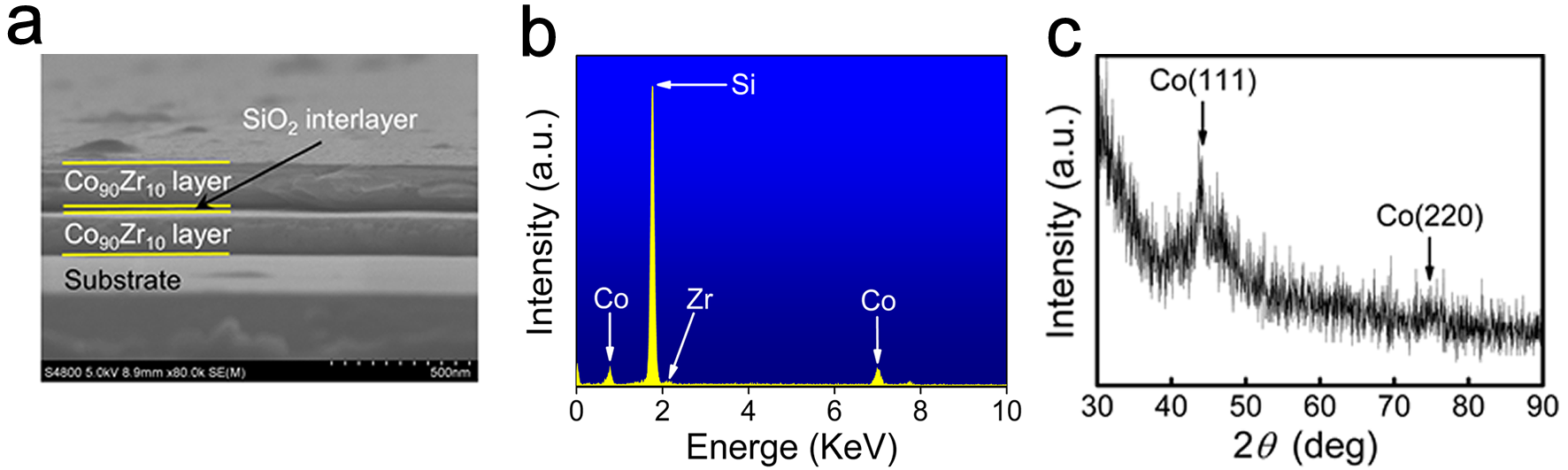


Supplementary Figure S2. a) SEM image of the cross section of the Co_90_Zr_10_/SiO_2_/Co_90_Zr_10_ FNF film. b) EDS result of the Co_90_Zr_10_ single layer. c) XRD pattern of the Co_90_Zr_10_ single layer.

To determine the structure and composition of the samples, the SEM image, energy dispersive X-ray spectroscope (EDS) and XRD pattern were measured and shown in Supplementary Fig. S2. Supplementary Fig. S2a shows the SEM image of the cross section of the Co_90_Zr_10_/SiO_2_/Co_90_Zr_10_ FNF film. The result shows clearly that the Co_90_Zr_10_/SiO_2_/Co_90_Zr_10_ FNF film fabricated in this work is sandwich like structure as it is designed. Moreover, the EDS result (shown in Supplementary Fig. S2b) reveals that the composition of the Co-Zr layer is about Co_90_Zr_10_. The Co(111) and Co(220) peaks shown in Supplementary Fig. S2c indicates that the Co in Co_90_Zr_10_ layer has the face-centered cubic (fcc) lattice. The two peaks around the Co(111) peak come from CoZr*_x_* alloy phase (Co_23_Zr_6_, Co_5_Zr, ect.) in the Co_90_Zr_10_ layer. The low intensity and the large full width at half maximum of the peaks imply that the Co_90_Zr_10_ layer has both crystalline and amorphous phases in it.

S3. Controlling the magnitude of the anisotropy field of each Co_90_Zr_10_ layer


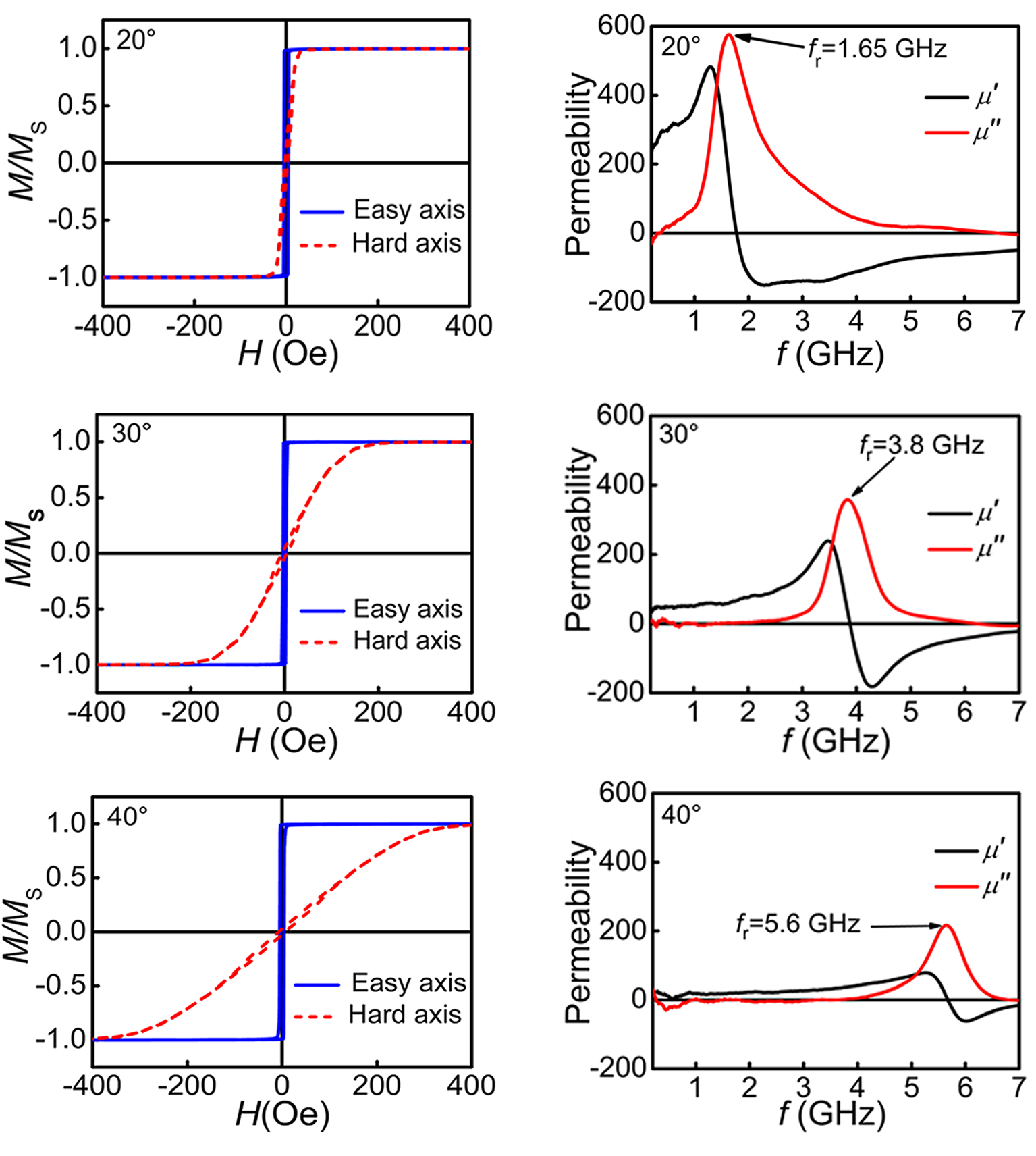


**Supplementary Figure S3.** In-plane magnetic hysteresis loops (left column) and permeability spectra (right column) of Co_90_Zr_10_ single layers that are fabricated by 20°, 30°, 40° oblique deposition.

Furthermore, as mentioned in the main text, the magnitudes of anisotropy fields of Co_90_Zr_10_/SiO_2_/Co_90_Zr_10_ FNF film are required to satisfy *H_K_*_1_ = *H_K_*_2_. So the controlling of the magnitude of anisotropy field in each Co_90_Zr_10_ layer was investigated. Supplementary Fig. S3 shows a series of magnetic hysteresis loops (left column) and permeability spectra (right column) of Co_90_Zr_10_ single layers those are fabricated by 20°, 30°, 40° oblique deposition. By calculating the data in Supplementary Fig. S3, the anisotropy fields of those three Co_90_Zr_10_ single layers are about 29 Oe, 152 Oe and 325 Oe, respectively. As a result, the magnitude of anisotropy field of the Co_90_Zr_10_ single layer is easily to control by the angle of oblique deposition. For each Co_90_Zr_10_(100 nm)/SiO_2_(10 nm)/Co_90_Zr_10_(100 nm) FNF film mentioned in the main text, the two Co_90_Zr_10_(100 nm) layers are fabricated in the same oblique angle, so the magnitudes of anisotropy fields of the two Co_90_Zr_10_(100 nm) layers are equal to each other, i.e. *H_K_*_1_ = *H_K_*_2_.

S4. Eliminating the exchange coupling between two Co_90_Zr_10_ layers


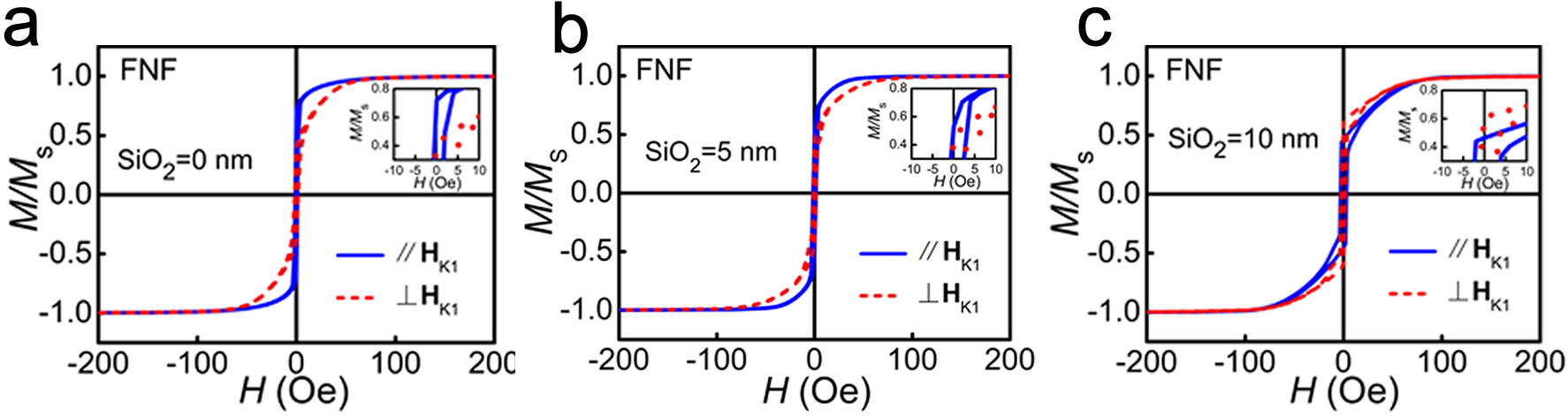


**Supplementary Figure S4.** In-plane magnetic hysteresis loops of Co_90_Zr_10_(100 nm)/SiO_2_(*x* nm)/Co_90_Zr_10_(150 nm) FNF films fabricated by 30° oblique deposition, thickness of SiO_2_ interlayers is a) 0 nm, b) 5 nm, c) 10 nm. **H***_K_*_1_ is the anisotropy field of Co_90_Zr_10_(100 nm) layer.

For Co_90_Zr_10_/SiO_2_/Co_90_Zr_10_ FNF film, it is necessary to eliminate exchange coupling between two Co_90_Zr_10_ layers by the SiO_2_ NM interlayer, so the direction and the magnitude of anisotropy field of each Co_90_Zr_10_ layer can be controlled separately. The thickness of the SiO_2_ NM interlayer in Co_90_Zr_10_/SiO_2_/Co_90_Zr_10_ FNF film was investigated. In order to see the elimination effect clearly, different thicknesses of FM1 and FM2 are used. Supplementary Fig. S4 shows the in-plane magnetic hysteresis loops measured along and perpendicular to **H***_K_*_1_ of the Co_90_Zr_10_(100 nm)/SiO_2_(*x* nm)/Co_90_Zr_10_(150 nm) FNF films fabricated by 30° oblique deposition. **H***_K_*_1_ is the anisotropy field of Co_90_Zr_10_(100 nm) layer, the thickness of SiO_2_ NM interlayer varies from 0 nm to 10 nm. If the exchange coupling between two Co_90_Zr_10_ layers were eliminated, the ratio of remanences measured along and perpendicular to H*_K_*_1_ should be 100/150 ≈ 0.67. According to the data in Supplementary Fig. S4, SiO_2_(10 nm) NM interlayer is able to eliminate the exchange coupling between two Co_90_Zr_10_ layers. Certainly, the SiO_2_ NM interlayer thicker than 10 nm still works.
